# Plasma concentrations of 5-fluorouracil and F-*β*-alanine following oral administration of S-1, a dihydropyrimidine dehydrogenase inhibitory fluoropyrimidine, as compared with protracted venous infusion of 5-fluorouracil

**DOI:** 10.1038/sj.bjc.6601224

**Published:** 2003-08-26

**Authors:** Y Yamada, T Hamaguchi, M Goto, K Muro, Y Matsumura, Y Shimada, K Shirao, S Nagayama

**Affiliations:** 1 Medical Oncology Division, National Cancer Center Hospital, 5-1-1 Tsukiji, Chuo-ku, Tokyo 104-0045, Japan; 2 Taiho Pharmaceutical Co., Ltd. Pharmacokinetics Research Lab, 224-2 Ebisuno, Hiraishi, Kawauchi-cho, Tokushima 771-0194, Japan

**Keywords:** dihydropyrimidine dehydrogenase, 5-FU, pharmacokinetics, S-1

## Abstract

The pharmacokinetics and pharmacodynamics of oral S-1, a dihydropyrimidine dehydrogenase (DPD) inhibitory fluoropyrimidine, were compared with those of protracted venous infusion (PVI) of 5-fluorouracil (5-FU). In all, 10 patients with gastric cancer received PVI of 5-FU at a dose of 250 mg m^−2^ day^−1^ for 5 days. After a washout period of 9 days, the patients received two divided doses daily for 28 days. S-1 was administered orally at about 0900 and 1900 hours. The daily dose of S-1 in terms of tegafur was 80 mg day^−1^ in patients with a body surface area (BSA) of <1.25 m^2^, 100 mg day^−1^ in those with a BSA of ⩾1.25 m^2^ to <1.5 m^2^, and 120 mg day^−1^ in those with a BSA of ⩾1.5 m^2^. Plasma concentrations of 5-FU and F-*β*-alanine (FBAL) were measured for pharmacokinetic analysis, and the plasma uracil concentration was monitored as a surrogate marker of DPD inhibition (pharmacodynamic analysis) in the same patients on days 1–5 of PVI of 5-FU and on days 1–5 of oral S-1. The area under the curve (AUC_0–10 h_) of 5-FU on day 5 was 728±113 ng h ml^−1^ for PVI of 5-FU and 1364±374 ng h ml^−1^ for S-1. The median 5-FU PVI : S-1 ratio of the AUC_0–10 h_ of 5-FU was 1.9. The AUC_0–10 h_ of FBAL on day 5 of PVI of 5-FU was 9465±3225 ng h ml^−1^, AUC_0–10 h_, as compared with 1725±605 ng h ml^−1^ on day 5 of S-1 treatment. The AUC_0–10 h_ of uracil on day 5 was 252±60 ng h ml^−1^ with PVI of 5-FU and 12 582±3060 ng h ml^−1^ with S-1. The AUC_0–10 h_ of FBAL was markedly lower and plasma uracil concentrations were significantly higher for S-1 than for PVI of 5-FU, clearly demonstrating the effect of DPD inhibition.

Continuous protracted intravenous infusion (PVI) of 5-fluorouracil (5-FU) has a higher response rate, less frequent haematologic toxicity (mainly neutropenia), and more frequent hand–foot syndrome (HFS) than bolus injection of 5-FU in patients with metastatic colorectal cancer (The Meta-Analysis Group in Cancer, 1998). Hand–foot syndrome and stomatitis each occur in about 23% of patients given 5-FU by PVI, whereas grade 3 leukopenia develops in only 1%. Most patients who receive 5-FU by PVI have no severe toxicity; the dose-limiting toxicity (DLT) is HFS (Lokich *et al*, 1989). Previous clinical trials have shown that PVI of 5-FU is one of the most effective and safest regimens. However, PVI requires a central venous catheter as well as an ambulatory pump, which is expensive and may lead to complications, and adversely affect patients' quality of life.

Oral fluoropyrimidine derivatives have been developed to circumvent the problems associated with PVI of 5-FU. S-1 is one such derivative that combines tegafur with two modulators of 5-FU metabolism, 5-chloro-2,4-dihydroxypyridine (CDHP), a reversible inhibitor of dihydropyrimidine dehydrogenase (DPD), and potassium oxonate, in a molar ratio of 1 : 0.4 : 1. Tegafur, an oral prodrug of 5-FU, is gradually converted to 5-FU and rapidly metabolised by DPD in the liver. The DPD-inhibitory activity of CDHP is 180-fold higher than that of uracil, confirmed to be an effective DPD inhibitor in the form of uracil/tegafur (UFT) *in vitro*. Potassium oxonate is an orotate phosphoribosyl transferase inhibitor that is distributed primarily to the gastrointestinal tract. This component of S-1 decreases the incorporation of 5-fluorouridine triphosphate into RNA in the gastrointestinal mucosa and reduces the incidence of diarrhoea.

F-*β*-alanine (FBAL) is a main metabolite of 5-FU. F-*β*-alanine and fluorocitrate are thought to cause the cardiotoxic and neurotoxic effects of 5-FU by inhibiting the tricarboxylic acid cycle (Koenig and Patel, 1970; Okeda *et al*, 1990; Robben *et al*, 1993; Diasio, 1998; Kuwata *et al*, 2000; Kato *et al*, 2001). The CDHP component of S-1 inhibits DPD, the rate-limiting enzyme in the catabolic pathway of 5-FU. Consequently, the plasma FBAL concentration after oral administration of S-1 is significantly lower than that after PVI of 5-FU. However, information on plasma FBAL concentrations in patients given 5-FU remains scant (Van Kuilenberg *et al*, 2001).

The objectives of this study were to investigate differences in pharmacokinetics (PK) and pharmacodynamics (PD) between oral S-1 and PVI of 5-FU and to examine the DPD inhibitory activity of S-1.

## MATERIALS AND METHODS

### Anticancer drugs

S-1 capsules combining tegafur, CDHP, and potassium oxonate in a molar ratio of 1 : 0. 4 : 1 (Taiho Pharmaceutical Co. Ltd., Tokyo, Japan) were used. Each capsule contained 20 or 25 mg of tegafur. 5-fluorouracil was obtained from Kyowa Hakko Co., Ltd. (Tokyo, Japan).

### Patients

In all, 10 consecutive patients with histologically proven metastatic gastrointestinal cancer were enrolled into this pharmacokinetic study. All patients were between 20 and 75 years of age and had an Eastern Cooperative Group (ECOG) performance status of 0–2, adequate baseline bone marrow (WBC count 3500 *μ*l^−1^ or more and platelet 100 000 *μ*l^−1^ or more, hepatic (serum bilirubin level 1.5 mg dl^−1^ or less, and serum GOT and GPT 40 U l^−1^ or less), and renal (serum creatinine level 1.2 mg dl^−1^ or less) function, and a life expectancy of at least 12 weeks.

Patients were excluded if they had symptomatic brain metastasis or other serious concurrent disease. This study was approved by the institutional review board of the National Cancer Center Hospital. Written informed consent was obtained from each patient before enrolment.

### Study design

All 10 patients received 5-FU by PVI at a rate of 250 mg m^−2^ day^−1^ via a peripheral vein. Infusion was carried out with the use of a battery-operated pump (STC-508, Terumo Co., Ltd., Tokyo, Japan) for five consecutive days. The assigned daily dose of 5-FU was diluted in 500 ml of normal saline for infusion. After a washout period of 9 days, patients received S-1 in two divided doses daily for 28 consecutive days. S-1 was administered orally within 1 h after breakfast and supper, at about 0900 and 1900 hours The daily dose of S-1 in terms of tegafur was 80 mg day^−1^ in patients with a body surface area (BSA) of <1.25 m^2^, 100 mg day^−1^ in those with a BSA of ⩾1.25 m^2^ to <1.5 m^2^, and 120 mg day^−1^ in those with a BSA of ⩾1.5 m^2^.

### Blood and urine sample collection

On days 1 and 5 of 5-FU PVI, blood samples were taken at 0 (before administration), 2, 6, and 10 h after the start of the infusion (0900 hours in all patients). On days 2–4, one blood sample was obtained at 0900 hours before the start of the next 24-h cycle. During oral administration of S-1, blood samples were taken on day 1 before the first dose of the day and at 1, 2, 4, 6, and 10 h. On days 2–4 of S-1 treatment, blood samples were taken before administration in the morning and at 4 and 10 h. On day 5, blood samples were obtained at 0, 2, 4, and 10 h. Peripheral blood samples (6 ml per sampling time) were collected in heparinised tubes and centrifuged at 1000 **g** for 15 min at 4°C. The plasma was stored at −20°C until analysis. Urine was collected from 0 to 24 h after the start of drug administration on day 1 of each treatment and refrigerated during the collection period.

### Assay of 5-FU, uracil, and FBAL

Analysis of 5-FU and uracil was carried out as described by Matsushima *et al* (1997), with minor modification. 5-Fluorouracil and uracil were extracted with ethyl acetate after washing with dichloromethane and were subjected to a reaction to induce their trimethylsilyl derivatives. 5-Fluorouracil and uracil were analysed by electron impact ionisation gas-chromatography/mass-spectrometry (GC–MS). For analysis of 5-FU by GC–MS, stable isotopes were used as internal standards. The measurable ranges of plasma or urine levels were 1–400 ng ml^−1^ for 5-FU and 5–2000 ng ml^−1^ for uracil. For analysis of FBAL, plasma was deproteinised with ethanol and washed with dichloromethane. F-*β*-alanine was treated with 2,4-dinitrofluorobenzene to derive its dinitrophenyl ester, and the reaction product was extracted with dichloromethane under acidic conditions. The reaction product was separated by high-pressure liquid chromatography (LC) with a reversed-phase column, and LC/tandem-mass spectrometry was performed with the use of negative ion-electron spray ionisation. DL-norvaline was used as an internal standard. The measurable range of plasma or urine levels was 5–2000 ng ml^−1^ for FBAL.

### Pharmacokinetics

Maximum plasma concentrations (*C*_max_) were determined from the observed highest concentration after treatment with PVI of 5-FU or oral S-1. The area under the curve (AUC) was calculated from 0 to 10 h (AUC_0–10 h_) on days 1 and 5 of each treatment according to the trapezoidal rule, using a WinNonlin program (Ver. 3.1, Pharsight Co., Mountain View, CA, USA). The same program was used to simulate plasma 5-FU concentrations during treatment with S-1. AUCs were calculated on the basis of data obtained at the following time points; 0, 1, 2, 4, 6, and 10 h for S-1 on day 1; 0, 2, 4, and 10 h for S-1 on day 5; and 0, 2, 6, and 10 h for PVI of 5-FU on days 1 and 5.

### DPD activity

DPD activity was determined by a catalytic assay as described by Ikenaka *et al* (1979). Blood samples were collected from patients before the start of 5-FU PVI, at about 0900 hours. Peripheral blood mononuclear cells (PBMC) prepared from about 10 ml of blood were thawed and placed in 250 *μ*l of a homogenised buffer containing 10 mM Tris-HCl, 1 mM EDTA, and 0.5 mM dithiothreitol, pH 7.4. The PBMCs were then homogenised by sonication, and the homogenate was centrifuged at 105 000 **g** for 60 min (4°C). The supernatant fraction was collected as the enzyme source and was frozen and stored at −80°C until analysis.

## RESULTS

Between April and October 2000, 10 male patients were enrolled. Their median age was 66 years (range, 45–75 years). All 10 patients had metastatic gastric carcinoma and eight had undergone gastrectomy. Two patients had previously received chemotherapy (irinotecan plus mitomycinC in 1, and cisplatin plus irinotecan in the other). The median body surface area (BSA) was 1.52 m^2^ (range, 1.38–1.85 m^2^). The initial doses of S-1 were 100 mg body^−1^ day^−1^ in four patients and 120 mg body^−1^ day^−1^ in six patients. The median actual dose of S-1 in the 10 patients was 71.6 mg m^−2^ day^−1^ (range, 64.8–80.0 mg m^−2^ day^−1^) (Table 1

**Table 1:** Pharmacokinetic parameters of 5-FU, uracil, and FBAL in 10 patients receiving PVI and S-1

	**5-FU**		**Uracil**		**FBAL**	
	**Day 1**	**Day 5**	**Day 1**	**Day 5**	**Day 1**	**Day 5**
*C*_max_ (ng ml^−1^)						
PVI	93±24	93±13	24±7	30±9	858±13	1157±350
S-1	144±33	230±69	1262±355	1559±409	100±69	198±74
*P*-value^a^	0.004	<0.001	<0.001	<0.001	<0.001	<0.001

AUC (ng hr ml^−1^)						
PVI	733±164	728±113	200±60	252±60	6267±113	9465±3225
S-1	857±209	1364±374	9033±2176	12582±3060	767±395	1725±605
*P*-value^a^	0.181	<0.001	<0.001	<0.001	<0.001	<0.001

FBAL=F-*β*-alanine.

a Paired *t*-test.

).

### 5-FU, uracil, and FBAL PK

**Figure 1:**
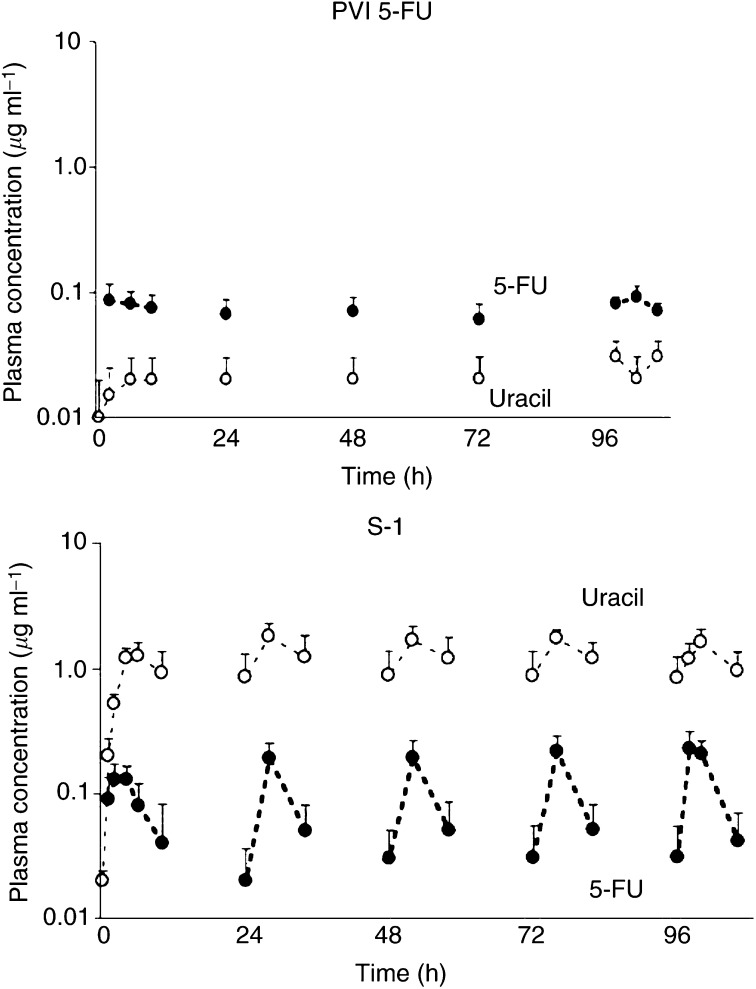
Plasma concentrations of 5-FU and uracil *vs* time after PVI of 5-FU and after oral S-1. Closed and open circles show the concentration of 5-FU and concentration of uracil, respectively. The left and right graphs show data for PVI of 5-FU and for S-1, respectively. Data are presented as means; bars, s.d.

The plasma concentrations of 5-FU, uracil, and FBAL after administration of PVI 5-FU and oral S-1 in the 10 patients on days 1–5 are shown in Figures 1

and 2

**Figure 2:**
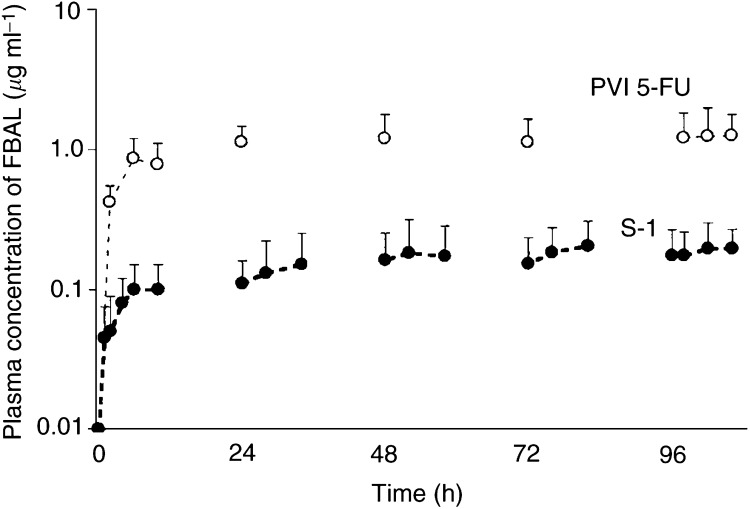
Plasma concentration of FBAL *vs* time after PVI of 5-FU and after oral S-1. Closed and open circles show the plasma concentration of FBAL after S-1 and PVI of 5-FU, respectively. Data are presented as means; bars, s.d.

. The plasma 5-FU concentration reached a steady state 2 h after the start of PVI. This steady state was maintained throughout the 5 days. During treatment with S-1, the time to the peak plasma concentration of 5-FU was 2–4 h. The *C*_max_ of 5-FU after S-1 treatment on day 1 was lower than the *C*_max_ of 5-FU on days 2–5 of S-1 treatment. The plasma concentration of uracil during S-1 treatment was higher than the baseline value.

The *C*_max_ and AUC_0–10 h_ of 5-FU in plasma during S-1 treatment were higher than the steady-state concentration and AUC_0–10 h_ of 5-FU in plasma during PVI of 5-FU. On day 5, the median AUC_0–10 h_ and *C*_max_ of 5-FU with S-1 were 1.9- and 2.5-fold greater than the respective values with PVI of 5-FU.

The plasma FBAL concentration during S-1 treatment was markedly lower than that during PVI of 5-FU. On day 5, the AUC_0–10 h_ of FBAL with PVI of 5-FU was 5.5-fold greater than that with S-1.

On day 1 of PVI of 5-FU, 1.2±0.7% (range, 0.3–2.3%) and 39.2±11.3% (range, 15.9–52.9%) of the administered dose was excreted in urine as 5-FU and FBAL, respectively. On day 1 of treatment with oral S-1, 7.3±2.2% (range, 3.9–11.0%) and 21.7±6.6% (range, 13.0–35.0%) of the administered dose were excreted in urine as 5-FU and FBAL, respectively.

The mean DPD activity of PBMC was 226 pmol min^−1^ mg^−1^ (range, 128–492 pmol min^−1^ mg^−1^). There was no correlation between the DPD activity of PBMC and the plasma concentration or AUC_0–10 h_ of 5-FU.

## DISCUSSION

This study showed that the *C*_max_ and AUC_0–10 h_ of 5-FU during treatment with oral S-1, given at dose levels recommended on the basis of phase I and II trials, were about two times greater than the respective values during PVI of 5-FU. Our results suggest that the higher incidence of leukopenia and diarrhoea associated with S-1, as compared with PVI of 5-FU, may be attributed to the significantly higher *C*_max_ and AUC of plasma 5-FU (Thyss *et al*, 1986; Vokes *et al*, 1996; Van Groeningen *et al*, 2000). In the phase I trial with once daily administration schedule of S-1, pharmacodynamic analysis demonstrated a correlation between grade of diarrhoea and both *C*_max_ of 5-FU (*r*=0.57, *P*<0.05) and AUC of 5-FU (*r*=0.74, *P*<0.01) (Cohen *et al*, 2002).

The plasma concentration of FBAL, the main catabolite of 5-FU, was significantly lower during S-1 treatment than during PVI of 5-FU. The AUC_0–10 h_ of the plasma FBAL concentration on day 5 of PVI of 5-FU was five-fold higher than that on day 5 of treatment with S-1. The plasma uracil concentration increased during S-1 treatment. The *C*_max_ of uracil was attained 4 h after oral administration of S-1 and decreased at 10 h. These results provide evidence that CDHP reversibly inhibits DPD. Plasma levels of FBAL and uracil have also been measured in European phase I studies of S-1. Very low levels of FBAL were detected because of DPD inhibition in 4 patients given S-1 (Van Kuilenberg *et al*, 2001). This low level of FBAL in plasma may be responsible for the decreased incidence of neurotoxicity and cardiotoxicity associated with 5-FU. In fact, several phase I and II clinical trials of S-1 have reported no cardiotoxicity or neurotoxicity (Taguchi *et al*, 1997; Sakata *et al*, 1998; Sugimachi *et al*, 1999; Koizumi *et al*, 2000; Ohtsu *et al*, 2000; Cohen *et al*, 2002; Chollet *et al*, 2003; Hoff *et al*, 2003; Van den Brande *et al*, 2003).

Capecitabine is also an oral fluoropyrimidine derivative (Budman *et al*, 1998). When capecitabine was administered orally after meals at a dose of 1255 mg m^−2^, the AUC of the plasma FBAL concentration was 31 400 ng h ml^−1^Reigner *et al*, 1998). This is 3.4-fold greater than the AUC of FBAL during PVI of 5-FU and 18-fold higher than that during treatment with oral S-1. The AUC of plasma 5-FU after treatment with capecitabine was 698 ng hr ml^−1^, and the *C*_max_ of plasma 5-FU was 310 ng ml^−1^. The AUC of 5-FU was similar to that during PVI of 5-FU and about half that during treatment with S-1. The *C*_max_ of 5-FU was 3.3-fold higher than that during PVI of 5-FU and 1.3-fold higher than that during S-1 treatment. Thus, the AUC of the plasma FBAL concentration after oral capecitabine was much higher than that after oral S-1. In a randomised phase II trial of capecitabine in patients with metastatic colorectal cancer, the incidence of cardiotoxicity was 5% (five out of 108) (Van Cutsem *et al*, 2000). The treatment was discontinued in four patients because of chest pain, angina, or atrial fibrillation. Paresthesia occurred in 12% of the patients (Van Cutsem *et al*, 2000). The incidence of neurotoxicity was high, as compared with that during PVI of 5-FU or during treatment with S-1.

Capecitabine is associated with a high incidence of HFS, occurring in 45% of patients (Van Cutsem *et al*, 2000). The incidence of grade 3 HFS is 15–18% (Van Cutsem *et al*, 2000, 2001; Hoff *et al*, 2001). Hand–foot syndrome is also a major problem during PVI of 5-FU with a reported incidence of about 23% (Lokich *et al*, 1989). S-1 has a very low incidence of HFS (Taguchi *et al*, 1997; Sakata *et al*, 1998; Sugimachi *et al*, 1999; Koizumi *et al*, 2000; Ohtsu *et al*, 2000; Van Groeningen *et al*, 2000; Cohen *et al*, 2002; Hoff *et al*, 2003; Peters *et al*, 2003). Hand–foot syndrome has been observed in just eight out of 3808 mainly Asian patients (0.2%) treated with S-1, according to the safety database of the manufacturer of this compound, Taiho Pharmaceuticals. In addition, HFS occurred in 9–10% in European phase II trial; however, most of the cases were limited to grade 1–2 (Chollet *et al*, 2003; Van den Brande *et al*, 2003). Interestingly, a low incidence of HFS has also been reported for eniluracil, an irreversible DPD inhibitor, plus oral 5-FU (Mani *et al*, 2000). Only two of 55 patients (4%) treated with oral 5-FU plus eniluracil had mild HFS. These findings suggest that the degree of DPD inhibition may correlate with the frequency and grade of HFS. Although the mechanism of HFS remains unknown, it may occur secondarily to the formation of 5-FU catabolites. Owing to modulation at the DPD level, 5-FU catabolites are less likely to form during treatment with S-1 and oral 5-FU plus eniluracil, as compared with capecitabine. However, we have no direct evidence showing that FBAL causes HFS.

In conclusion, we found that oral S-1 at presently recommended doses produced a higher *C*_max_ and a greater AUC of plasma 5-FU than did PVI of 5-FU, with no elevation of the plasma FBAL concentration. During S-1 treatment, DPD inhibition by CDHP apparently resulted in lower plasma FBAL concentrations and higher plasma uracil concentrations than did PVI of 5-FU.
